# Increased winter drownings in ice-covered regions with warmer winters

**DOI:** 10.1371/journal.pone.0241222

**Published:** 2020-11-18

**Authors:** Sapna Sharma, Kevin Blagrave, Simon R. Watson, Catherine M. O’Reilly, Ryan Batt, John J. Magnuson, Tessa Clemens, Blaize A. Denfeld, Giovanna Flaim, Laura Grinberga, Yukari Hori, Alo Laas, Lesley B. Knoll, Dietmar Straile, Noriko Takamura, Gesa A. Weyhenmeyer

**Affiliations:** 1 Department of Biology, York University, Toronto, Ontario, Canada; 2 Department of Geography, Geology, and The Environment, Illinois State University, Normal, Illinois, United States of America; 3 Rutgers University, New Brunswick, New Jersey, United States of America; 4 Center for Limnology, University of Wisconsin-Madison, Madison, Wisconsin, United States of America; 5 Drowning Prevention Research Centre Canada, Toronto, Ontario, Canada; 6 Department of Ecology and Environmental Science, Umeå University, Umeå, Sweden; 7 Department of Sustainable Agro-ecosystems and Bioresources, Research and Innovation Centre, Fondazione Edmund Mach, San Michele all'Adige, Italy; 8 Department of Botany, The Latvian Museum of Natural History, Riga, Latvia; 9 Department of Physical and Environmental Sciences, University of Toronto Scarborough, Toronto, Ontario, Canada; 10 Institute of Agricultural and Environmental Sciences, Estonian University of Life Sciences, Tartu, Estonia; 11 Itasca Biological Station and Laboratories, University of Minnesota Twin Cities, Lake Itasca, Minnesota, United States of America; 12 Limnological Institute, University of Konstanz, Konstanz, Germany; 13 Lake Biwa Branch Office, Center for Environmental Biology and Ecosystem Studies, National Institute for Environmental Studies, Otsu, Shiga, Japan; 14 Department of Ecology and Genetics/Limnology, Uppsala University, Uppsala, Sweden; University of Nevada, Reno, UNITED STATES

## Abstract

Winter activities on ice are culturally important for many countries, yet they constitute a high safety risk depending upon the stability of the ice. Because consistently cold periods are required to form stable and thick ice, warmer winters could degrade ice conditions and increase the likelihood of falling through the ice. This study provides the first large-scale assessment of winter drowning from 10 Northern Hemisphere countries. We documented over 4000 winter drowning events. Winter drownings increased exponentially in regions with warmer winters when air temperatures neared 0°C. The largest number of drownings occurred when winter air temperatures were between -5°C and 0°C, when ice is less stable, and also in regions where indigenous traditions and livelihood require extended time on ice. Rates of drowning were greatest late in the winter season when ice stability declines. Children and adults up to the age of 39 were at the highest risk of winter drownings. Beyond temperature, differences in cultures, regulations, and human behaviours can be important additional risk factors. Our findings indicate the potential for increased human mortality with warmer winter air temperatures. Incorporating drowning prevention plans would improve adaptation strategies to a changing climate.

## Introduction

Winter activities on ice are a defining component of winter culture and identity in northern countries, contributing food through ice fishing, winter transportation on ice roads, and recreational activities, such as ice skating [1]. Winter ice fishing provides food, economic growth, and recreational opportunities for local communities. For example, the annual Brainerd Jaycees Ice Fishing Extravaganza in Minnesota contributes 1 million USD to the local economy [1]; 100,000 people attend traditional ice fishing events in remote China where the first fish caught sells for tens of thousands of dollars [2]; and 40% of the annual fish harvest in Lake Peipsi, Estonia, is from ice fishing [3]. In the Apostle Islands in Lake Superior, children rely on winter ice roads to attend school during the winter [4], whereas remote communities in Canada and Siberia depend on winter ice roads for access to resources and social networks (e.g. [5]). In regions where traditional hunting and fishing contributes to the livelihoods of some Indigenous groups, increased numbers of search and rescue events on ice have been associated with warmer winter air temperatures and poor ice conditions [6, 7]

Falling through the ice is one of the major risk factors associated with winter ice activities, resulting in fatal and non-fatal winter drownings. Globally, drowning is the third leading cause of unintentional injury deaths [8], and in northern countries, approximately 20% of fatal drownings in natural waters occur in winter when inland waters are typically frozen. Winter drownings are particularly hazardous, because cold shock and incapacitation can rapidly lead to unconsciousness, cardiac arrhythmia, and death [9, 10]. Non-fatal drownings (i.e., respiratory impairment from drowning that does not result in death) occur at least as often as fatal drownings, and carry substantial emotional, social, and economic costs because of the high likelihood of brain damage from submersion [11, 12]. To date, there has been no synthesis of winter drownings, despite the widespread health and socioeconomic consequences of these injuries and deaths. This information is critical given that winter weather and climate are currently undergoing rapid changes whereas human perceptions and behaviors are not [13].

The complex nature of interacting climate factors makes it difficult to predict the safety of ice conditions. Over the past century, lakes, rivers, and seas are experiencing earlier ice break-up, later ice formation, and shorter seasons of ice cover [14–16]. Moreover, there have been decreases in ice thickness and increases in the frequency of freeze-thaw events. In some winters, no ice cover may form at all [2, 14]. With warmer winters, ice cover will become more unpredictable, to the extent that a 1°C increase in average annual air temperatures could eliminate consistent lake ice cover for 100 million people who typically have access to a frozen lake [2]. Although air temperature is typically the most important driver of ice phenology in both freshwater and marine systems [17, 18], wind, snow cover, precipitation, solar radiation and water depth are also important determinants [17, 19]. Wind can inhibit solid ice formation by breaking up the ice when it first forms and facilitate the mechanical act of breakup in the spring [17]. Further, snow adds insulation to ice cover and can affect the thickness, type, and strength of ice beneath it [17, 19, 20]. In lakes, the strongest ice (black ice) is formed in clear and cold conditions, but in warm and wet winters there is relatively less black ice [19]. Thus, as warmer winters become more common with a higher frequency of freeze-thaw events [6], ice strength generally may be reduced overall, with potential implications for winter drownings.

Here, we explore whether there are more winter ice drownings in regions with warmer winters, because air temperature is a major factor for ice stability [16, 17]. We collated death records from coroner's offices, police stations, lifesaving societies and national statistics offices from 10 countries around the Northern Hemisphere where inland waters historically experience winter ice cover, specifically from Canada, Estonia, Finland, Germany, Italy, Japan, Latvia, Russia, Sweden, and the United States. We hypothesized that there would be more winter drownings in warmer winters because of unpredictable ice conditions and decreased ice stability [16, 18]. Further, we investigated the factors that contribute to patterns in drownings, such as timing, demographics, and the type of activity preceding drowning.

## Methods

This study is a multi-centered, multi-nation retrospective review of climate and fatal drowning event data for winter seasons, over 26 years. We used non-linear mixed effects models to characterize the relationship between winter air temperatures and winter drownings.

### Data acquisition

#### Drowning records

Drowning is defined as “the process of experiencing respiratory impairment from submersion/immersion in liquid." The outcomes can be fatal and non-fatal. Non-fatal drowning outcomes can be with or without morbidity [8]. We collected information on fatal drownings in winter from ten northern hemisphere countries that typically experience winter ice cover on inland waters. The period of data varied depending upon the country, from as early as 1991 to as recently as 2017. Data stewards from each country provided a dataset containing unique drowning cases based on multiple sources of information including coroners and medical examiners records; federal and regional agencies; and rescue organizations. We extracted data associated with the International Classification of Diseases (ICD) Diagnosis codes W69 (accidental drowning and submersion while in natural water), W70 (drowning and submersion following fall into natural waters), and W71 (and falling through the ice). ICD Diagnosis codes incorporate lakes, rivers, streams and open sea into ‘natural waters’.

Data were consolidated by month and region to conform to winter months and ice-covered regions. The Canadian North is represented by the Northwest, Yukon, and Nunavut Territories. Newfoundland, New Brunswick, Nova Scotia, and Prince Edward Island constitute the Canadian Atlantic. The Canadian Prairies consist of Alberta, Saskatchewan, and Manitoba. Italy is represented by the northern Trentino-South Tyrol region and Japan is represented by the Nagano prefecture. The United States is restricted to Alaska, Connecticut, Iowa, Maine, Massachusetts, Michigan, Minnesota, Montana, New Hampshire, New York, North Dakota, South Dakota, Vermont, and Wisconsin.

We were unable to acquire data on non-fatal drownings, which may occur at much greater numbers, and thus our results provide a conservative estimate of the overall consequences associated with winter drownings. Full details on how the data were acquired from each country are provided in S1 Table. Detailed demographic data were available for Minnesota, USA, which we used to explore the timing, activities, and ages associated with winter drownings through ice. Written ethics review and approval was obtained by the Human Participants Review Sub-Committee, York University’s Ethics Review Board and conforms to the standards of the Canadian Tri-Council Research Ethics guidelines. The ethics board did not require informed consent from the families/appropriate next of kin of those whose data were included in the study due to the retrospective nature of the study. Further, the data were anonymized before we accessed them.

#### Climate data

We acquired mean air temperature and precipitation data for each month corresponding to the time period and specific areas encompassed by the drowning records averaged for each region from the University of East Anglia’s Climatic Research Unit (CRU TS4.02). For example, for the United States, we averaged the winter climate data for the regions within the 14 northern states which contain lakes that typically experience winter ice cover. The climate data are available on a 0.5° latitude/longitude grid based on interpolations of air temperatures and precipitation measured at meteorological stations [21]. We calculated seasonal averages for winter temperature from the monthly means of December, January, February, and March to account for multicollinearity between monthly climate conditions.

#### Population data

We obtained population data for all countries from the CIA World Factbook website for 1990–2017. The US Bureau of the Census provides an annual estimate in July of the population for each country from population censuses and vital statistics registration systems (CIA World Factbook; https://www.cia.gov/library/publications/the-world-factbook/; https://www.gutenberg.org/wiki/CIA_World_Factbooks_(Bookshelf)). For provincial and state level population estimates, we obtained official national census data within each country. For the Trentino-South Tyrol region of Italy, we obtained the official national census data in 2001 and 2011 from the Italian National Statistics office (http://dati.istat.it/). For the Nagano prefecture in Japan, we obtained official census data every year for the period 2006 to 2018 (https://tokei.pref.nagano.lg.jp/). For Canada, we obtained annual provincial population numbers from Statistics Canada from 1992–2014 (https://www150.statcan.gc.ca/t1/tbl1/en/tv.action?pid=1710000501).

### Data analyses

To characterize the relationship between winter air temperatures and winter drowning, we developed a non-linear mixed effects model with country/region as a random effect and winter air temperatures as a fixed effect. We excluded Italy, Japan, and northern Canada from this analysis owing to the low number of drownings in these regions. For example, in Italy, there were only 5 winter drownings over the 12-year time series. Germany was also excluded from the model as its winters can have temperatures above 0°C. The data were modelled using an exponential function with a y-offset and run in both the nlme package in R 3.5 and non-linear least squares fitting in python.

To quantify in which month the most people drowned, we calculated a mean monthly average of winter drownings for each month over the complete time series for each region. For the detailed records from the Minnesota Department of Natural Resources, we generated a histogram of the number of drownings by age and vehicle type, including no vehicle, light vehicle (snowmobiles, all-terrain vehicle (ATV), utility terrain vehicle (UTV), off highway vehicle (OHV), and heavy vehicle (tractors, vans, trucks, sport utility vehicle (SUV) and other vehicles). The code and data used in the analysis are available at: www.github.com/kblagrave/winterdrownings/

## Results and discussion

**Fig 1:**
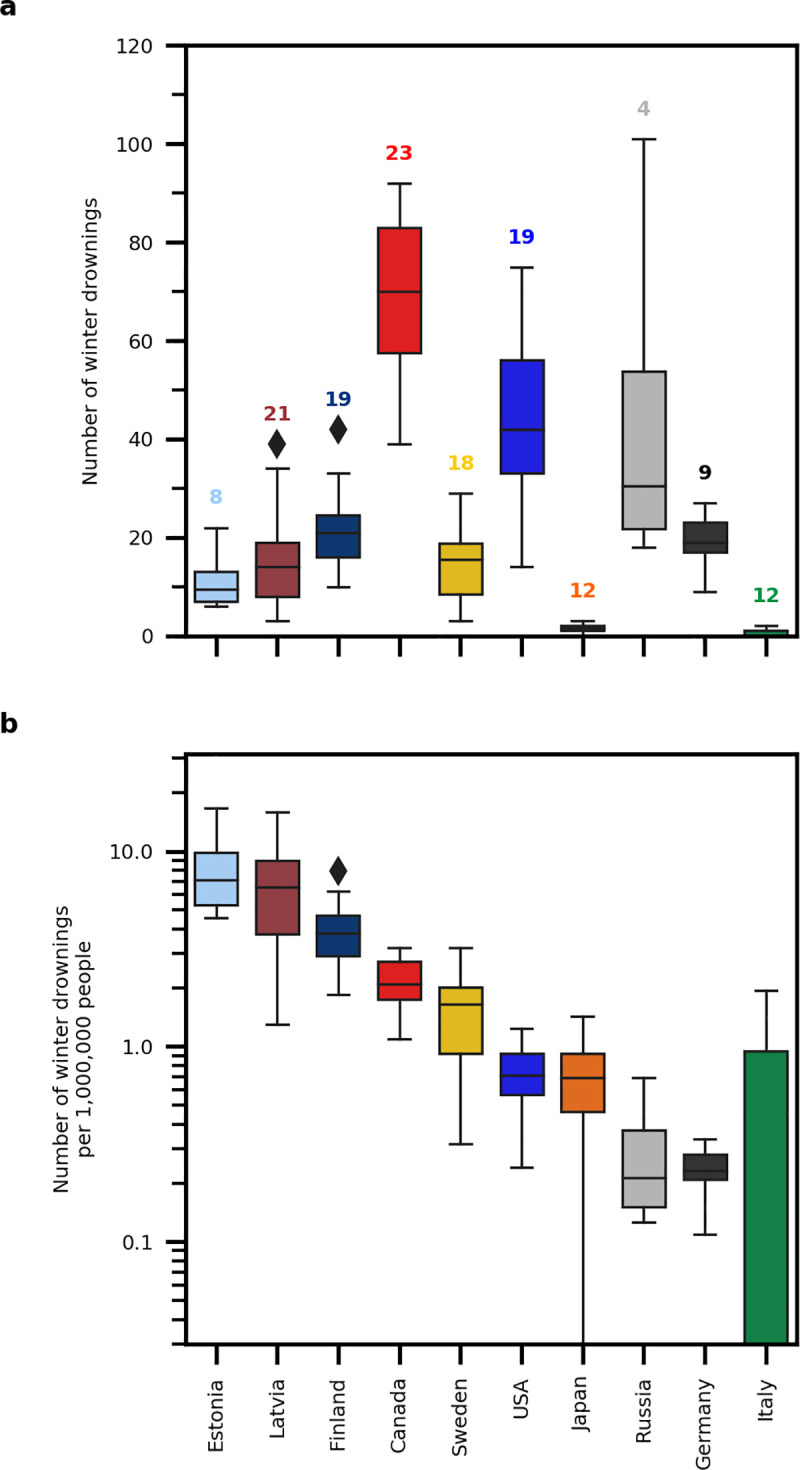
Boxplots summarizing winter drownings. Winter drownings in natural waters for 10 Northern Hemisphere countries. We collected winter drowning and human population data in regions within countries where inland waters typically experience winter ice cover. We collected winter drowning and human population data from all provinces/states in Canada, Estonia, Germany, Latvia, Finland, Russia, and Sweden. For Italy, we limited our data collection to the northern Trentino-South Tyrol region. For Japan, we collected data only in the Nagano prefecture. For the United States, we limited our data collection to 14 states in which inland waters typically experience winter ice cover, specifically Alaska, Connecticut, Iowa, Maine, Massachusetts, Michigan, Minnesota, Montana, New Hampshire, New York, North Dakota, South Dakota, Vermont, and Wisconsin. Number of drownings are presented as absolute numbers (a; linear axis) and numbers per capita (b; log axis). We present box plots with median quartile, range, and extremes during which we collected records for all countries. The numbers of years of data collected is included in the top plot above each country’s boxplot.

**Fig 2:**
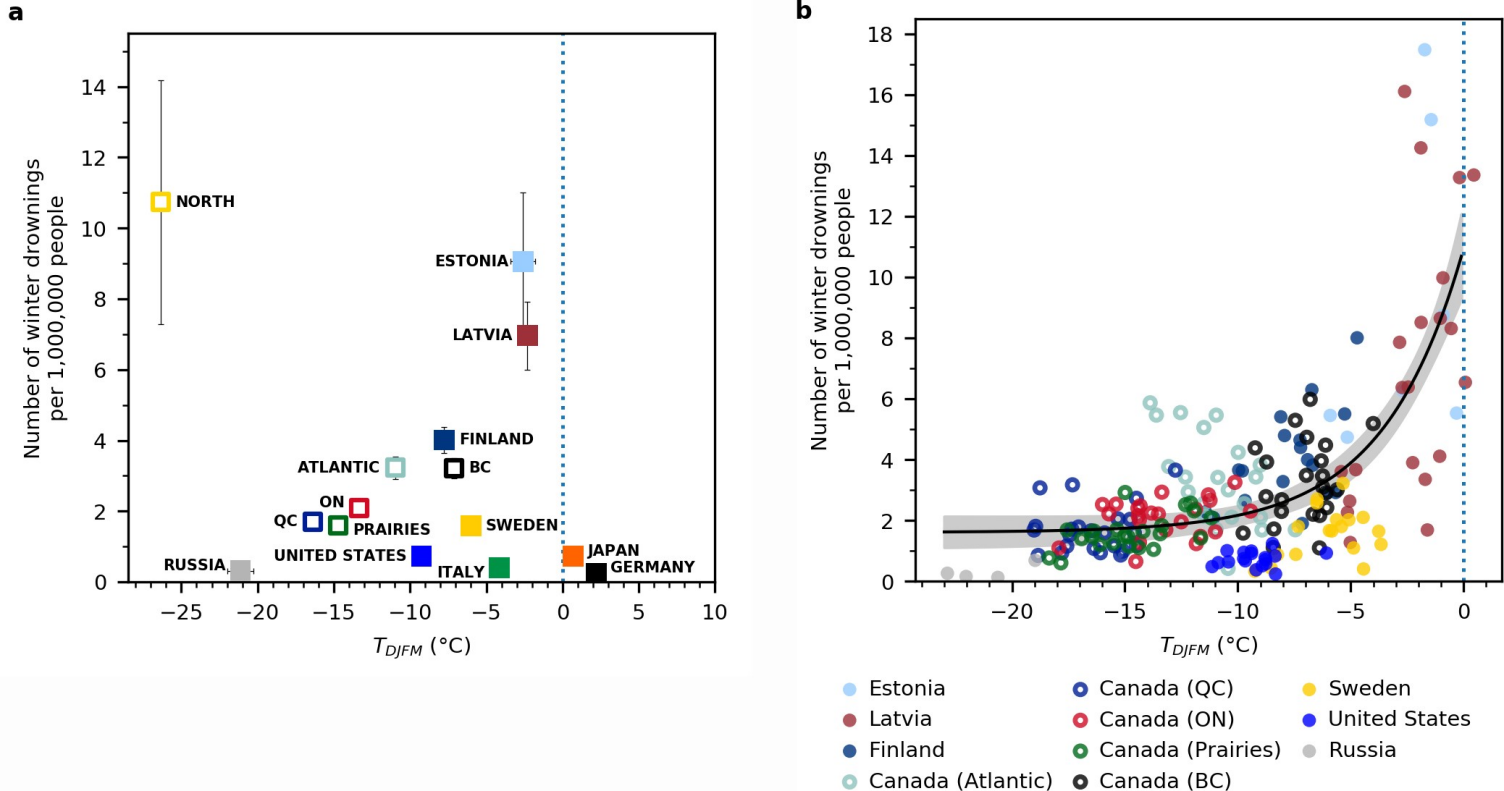
Winter drownings per capita as a function of mean regional winter temperature. a) Mean winter drownings per capita as a function of mean winter air temperature, with standard errors. Each point represents the region’s average over the years. b) Mean winter drownings per capita as a function of mean winter air temperature for regions with more than 10 drownings over their time series. Each point represents a different year. Canadian subregions are plotted as open circles. An exponential model with a y-offset (black line) explains 48% of the variation.

We documented over 4000 winter drownings (Fig 1), which contributed ~15–25%, but as high as 50% of total annual drownings in some countries (S1 Fig). A non-linear fixed effects model based on an exponential function revealed that winter air temperatures explained 48% of the variation in winter drownings across countries (Winter Drownings = 1.5 + 9.3*e^(0.4*DJFM Air Temperature); p<0.001; Fig 2A). A non-linear mixed effects model with country added as a random effect did not explain significantly more variation. At winter air temperatures below -10°C, the number of winter drownings was relatively low and similar across countries and regions (Fig 2). The likelihood of drowning is lower with cold winter air temperatures because lower winter air temperatures are associated with increased ice thickness [6]. However, we found one exception in northern Canada, where a very high number of individuals drowned per capita despite very cold winter conditions. (Fig 2B). We suggest that in northern Canada and Alaska, Indigenous communities may be especially vulnerable to higher rates of drownings because of their reliance on natural waters for food and transportation, which results in increased exposure time [10, 22].

Generally, winter drownings rapidly increased as warmer winter air temperatures approached 0°C (Fig 2A). At winter air temperatures between -10°C and -5°C, the variability of drownings among countries began to increase substantially (Fig 2). As winter air temperatures approached 0°C, the number of drownings was five times higher and varied by an order of magnitude among countries (Fig 2). Ice conditions in freshwater are more likely to become unstable above -5°C until it melts at 0°C. However, once winter air temperatures are above 0°C, when lakes are less likely to be consistently frozen throughout the winter [2, 23], winter drownings per capita were close to 0 in countries such as Germany and Japan (Fig 2A). Clark et al. documented a similar exponential threshold relationship with air temperatures in northern Canada [6]. The probability of search and rescue events peaked at minimum daily air temperatures of -3°C, which was associated with thinner ice and degrading ice conditions [6]. Search and rescue events declined above temperatures of 0°C because of lack of consistent ice cover [6]. Moreover, we suggest that the wide variation in drownings between -5°C and 0°C (Fig 2A) may reflect differences in cultural norms among countries. For example, ice fishing is very common in Estonia and Latvia where winter drownings were amongst the highest per capita. Conversely in Italy, where winter drownings are close to 0, ice fishing and snowmobiling are prohibited by local laws.

**Fig 3:**
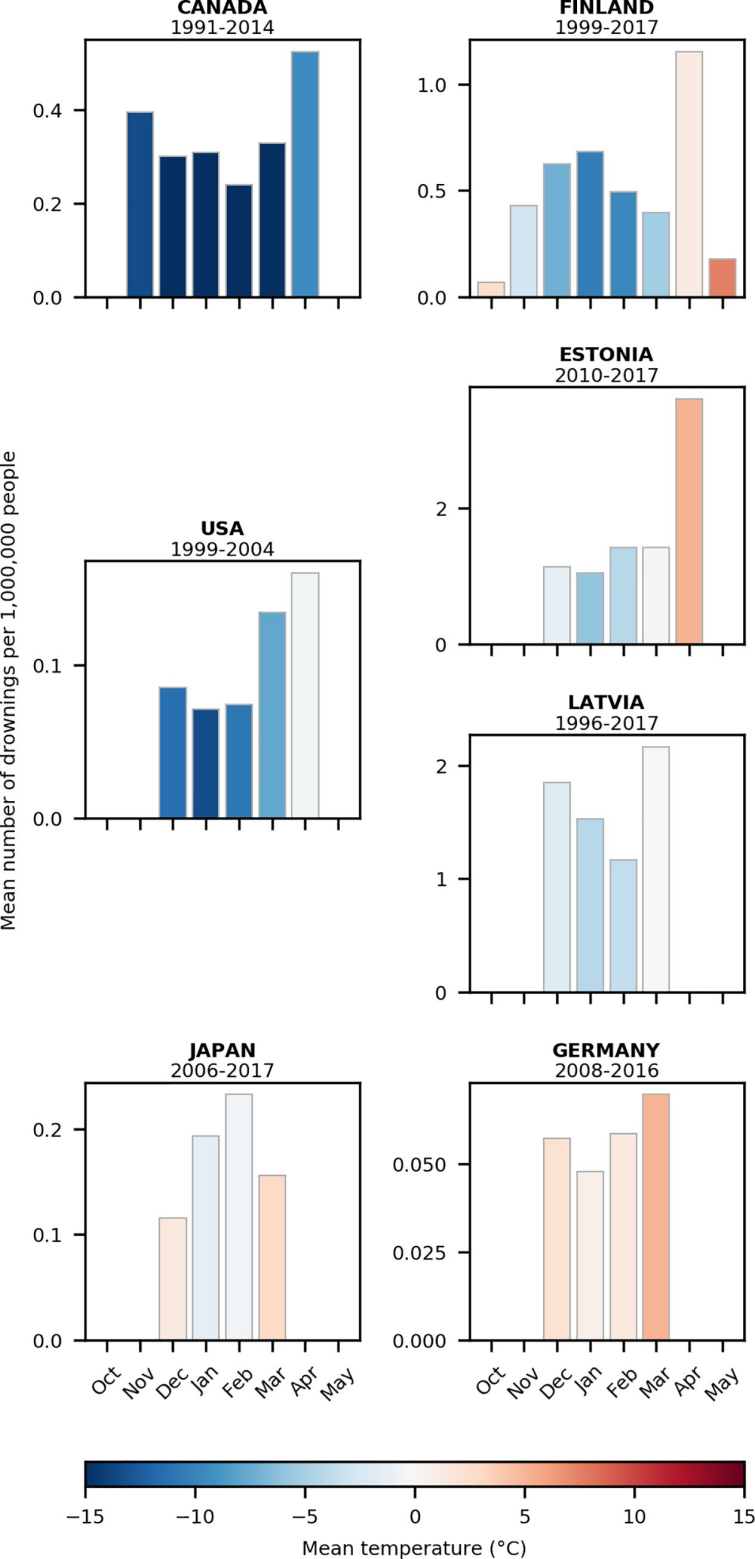
Winter drownings per capita by month. Monthly winter drownings are presented on the right for European countries and on the left for North America and Asia. Monthly means are calculated for each country using the full extent of the time series of drowning data; bar plot colours reflect the mean monthly temperatures by month for each country. Years are indicated above each plot. Note: each graph is scaled differently and Japan refers specifically to the Nagano prefecture.

Winter drownings were more likely to occur towards the end of winter, generally coinciding with periods when ice is more likely to be weak as air temperatures warm and the sun is higher in the sky (Fig 3). Most drownings occurred in the last month of winter: March for Latvia and Germany, and April for Canada, Finland, Estonia, and the United States (Fig 3). In Canada and Finland, we found that the number of drownings were significantly higher in April relative to other months (p<0.05 based on Kruskal-Wallis and post-hoc pairwise Mann-Whitney tests). In Latvia and USA, countries with fewer years with monthly time series, the greatest number of drownings also occurred in the last month of winter (Kruskal-Wallis test; Latvia p = 0.12; USA p = 0.11). Under-ice melting constitutes a major factor for the weakness of ice in the late winter and early spring and increases the danger of drowning [24]. The structural integrity of the ice in late winter and early spring decreases owing to a combination of snow melt, increase in light transmission through the ice, increase in solar radiation per unit area, increased freeze-thaw events during a season, and ice melting from below [24–26]. Early spring, and specifically April, is a higher-risk period because warmer temperatures and longer daylight hours encourage people to spend time outside, while at the same time melting lake ice becomes weaker [6, 27, 28]. Similarly, spring presented a higher risk of search and rescue events on ice when the ice is less thick and unstable [6]. Careful tracking of ice thickness and stability and providing this information to the public during ice formation and breakup could reduce drownings [29].

**Fig 4:**
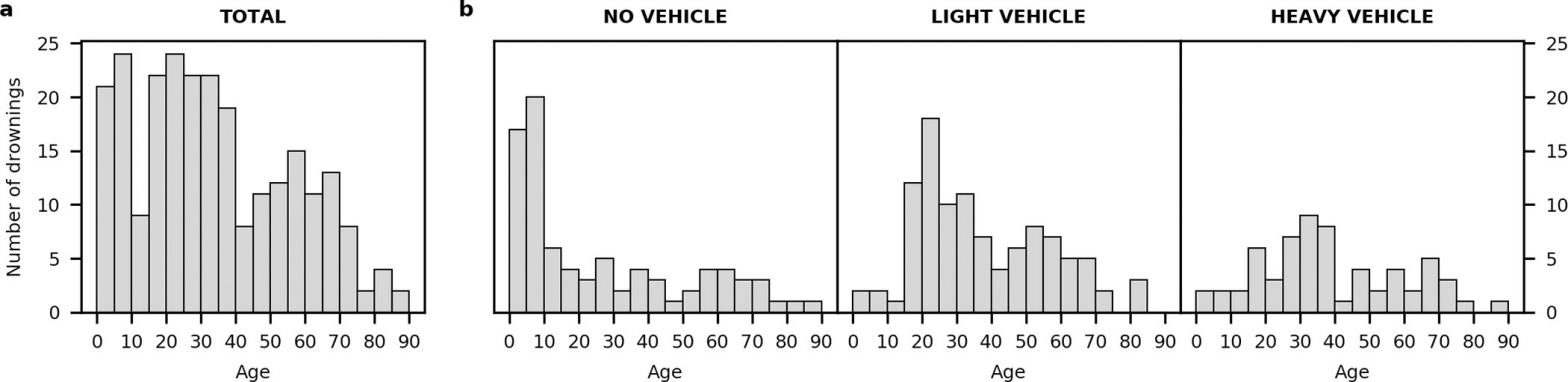
Ages and the type of vehicle used by the victims preceding drowning. Distribution of winter drowning deaths in Minnesota, USA, from 1976 to 2018 (a) total by age and (b) by vehicle type by age. No vehicle is for those walking, skiing, and skating. Light vehicles are those riding snowmobiles, ATVs, UTVs, and OHVs. Heavy vehicles are for those riding cars, trucks, tractors, vans, SUVs and working with other large equipment.

As a case study, we used detailed data from Minnesota, USA to illuminate additional risk factors for winter drowning, including providing details on the age and activity of the victim at time of drowning. The most vulnerable age groups for winter drowning were children and young adults (Fig 4A). Children under 9 years old accounted for 44% of drowning fatalities that did not involve a vehicle (Fig 4B). Most children drowned while playing or skating on the ice, and the risk of drowning was exacerbated by curiosity, inadequate supervision, and a lack of risk-awareness and water-safety education [22, 30]. Individuals from 15–39 years old were as vulnerable to drowning through ice as the youngest children (Fig 4A). These young to middle-aged individuals were amongst the highest-risk groups because they spent more time on the ice fishing and can engage in riskier behaviour [29, 31, 32]. Winter ice activities are frequently associated with alcohol consumption, which increases risk-taking behaviour and is a major contributing factor to drowning deaths [10, 27, 32, 33].

In addition to victim’s age, certain activities were more closely associated with drowning. Drowning while driving a light vehicle was more common than drowning while driving a heavy vehicle owing to the popularity of snowmobiles (Fig 4B). Specifically, young adults between the ages of 20–24 years old were the most vulnerable to drowning while operating a light vehicle, including snowmobiles (Fig 4B). Snowmobiles falling through the ice contributed the greatest number of drownings in Minnesota from 1976–2018. Snowmobiling is one of the most dangerous recreational activities in multiple states in the United States [29, 34], Canada [10], Sweden [28], and Finland [27], with the highest percentage of fatalities occurring on lake ice. Insufficient training, lack of regulations or challenges of enforcing regulations when they do exist, and high rates of impaired driving increase risk-taking behaviour on snowmobiles, such as driving at high speeds and over patches of open water [10, 28].

Our results underestimate the true consequences of through-ice drownings as we were unable to collect data on non-fatal drownings. Cold-water immersion is associated with unique and rapid physiological consequences that that can increase the likelihood of drowning. Humans immediately experience the cold shock response (gasping and uncontrolled breathing) for up to 2 minutes, and cold incapacitation which increases from 2 to 20 minutes; this can cause drowning very quickly. If the airway is protected during immersion, mild hypothermia occurs after 30–60 minutes, and unconsciousness, cardiac arrhythmia and death can occur after 2 or more hours; this will occur earlier with children due to their decreased body mass [9, 10]. Survival is hampered by the challenges of getting out of cold water onto the ice surface quickly, contributing to increased cold-water exposure. Among non-fatal cold-water drownings where children suffered cardiac arrest, only 10% of children had good neurological outcomes and only 27% of children were alive after one year [35]. Risks of poor neurobehavioral outcomes are high; cognitive functioning was significantly below average for 72% of children [36]. Thus, the impending consequences of changes in ice conditions extend beyond fatal drownings, as non-fatal drownings could also increase [6, 12].

Drowning requires immediate intervention, so prevention strategies are more effective for reducing drowning than rescue or treatment [8] Strong ice safety legislation and enforcement could reduce winter drownings in northern countries. In countries where drowning events are lowest, such as Italy, local laws and regulations prohibit ice fishing and snowmobiling; other activities such as skating on frozen lakes are often limited until local authorities deem the ice to be safe. However, legislation is unlikely to have a significant impact where ice access is associated with unregulated livelihood activities, such as in remote communities in the Canadian North [37]. The Russian Emergency Situation Ministry advises rules of conduct and behaviour on ice, including avoiding ice at night, while intoxicated, or in large groups [38] Swimming lessons with water safety instruction reduces drownings amongst children [8], and thus, including an ice safety component in water safety training provided to all children in northern countries could reduce winter drownings. The use of safety equipment, such as ice picks, flotation devices, hypothermia protective clothing, and safety education courses could further reduce winter drownings [10]. For example, in Wisconsin, snowmobile safety education courses and decreasing the nightly speed limit to 55 mph (~90 km/hr) may be associated with the reduced number of snowmobile drowning deaths [34].

The potential for increased winter drownings is a previously unrecognized consequence of warmer winters. Excess winter deaths have been difficult to correlate to cold temperatures because of the influence of seasonal factors such as influenza, respiratory infections, and cardiac risk [39]. However, our results suggest that given the inverse relationship between drownings and air temperature below 0°C, down to -10°C, winter drownings may increase in many countries across the Northern Hemisphere until a threshold is crossed where lakes no longer freeze, at which point drownings decrease. If average winter air temperatures are above 0°C in a particular year, we would expect winter drownings to decrease to near zero in any region, including Estonia, Latvia, Italy, and Germany. Our findings regarding higher winter mortality in warmer winters are consistent with projections for increased winter search and rescue events in a warming climate [6].

## Conclusions

Our results suggest that milder winter air temperatures will have implications for winter drownings in countries that have historically had ice cover on inland waters. The complex nature of changing winters including warming temperatures, rain on snow, and freeze-thaw events could decrease the stability of ice [2, 20, 40], suggesting that the risk of winter drowning may increase until lakes become completely ice-free. Human lives could be saved if considerations of air temperatures integrated throughout the winter were included in individual decision-making and risk calculations on the ice. For example, Cree hunters use observations of air temperatures and precipitation to continually evaluate inland ice conditions throughout the winter when implementing ice safety and security programs [41]. To empower individuals to take adaptive actions, stakeholder organizations such as life-saving societies, government agencies, Indigenous, and other local communities are vital for increasing awareness of the impacts of climate change on ice safety. Our results have widespread implications for public health education in countries where populations have historically not needed to pay attention to winter conditions. The rapid rate of winter warming and its relationship to loss of human life highlights the urgent need to incorporate interannual variation in climate into everyday decision-making in an effort to limit further tragedies.

## Supporting information

S1 Fig Boxplots summarizing drownings. Winter drownings are shown as a percentage of total annual drowning deaths with median quartile, range, and extremes for the time period during which we collected records for all countries. The number of years of data collected with monthly drowning data is included above the box plot for each county.
(DOCX)

S1 Table Detailed information on drowning data sources. For each of the 11 countries, we provide details on the types, sources and time periods of data that we used in this study. ICD Diagnosis codes are as follows: W69 (accidental drowning and submersion while in natural water), W70 (drowning and submersion following fall into natural waters), and W71 (and falling through the ice).
(DOCX)

## References

[1] Knoll LB, Sharma S, Denfeld BA, Flaim G, Hori Y, Magnuson JJ, et al Consequences of lake and river ice loss on cultural ecosystem services. Limnol. Oceanogr. Lett. 2019; 4: 119–131.

[2] Sharma S, Blagrave K, Magnuson JJ, O’Reilly CM, Oliver S, Batt RD, et al Widespread loss of lake ice around the Northern Hemisphere in a warming world. Nat. Clim. Change 2019; 9: 227–231.

[3] Orru K, Kangur K, Kangur P, Ginter K, Kangur A. Recreational ice fishing on the large Lake Peipsi: socioeconomic importance, variability of ice-cover period, and possible implications for fish stocks. Est. J. Ecol. 2014; 63: 282–298.

[4] Magnuson JJ, Lathrop RC. Lake ice: winter, beauty, value, changes, and a threatened future. LakeLine 2014; 43: 18–27.

[5] Hori Y, Cheng VYS, Gough WA, Jien JY, Tsuji LJS. Implications of projected climate change on winter road systems in Ontario’s Far North, Canada. Clim. Change 2018; 148: 109–122.

[6] Clark DG, Ford JD, Berrang-Ford L, Pearce T, Kowal S, Gough WA. The role of environmental factors in search and rescue incidents in Nunavut, Canada. Public Health 2016; 137: 44–49. 10.1016/j.puhe.2016.06.003 27423419

[7] Durkalec A, Furgal C, Skinner MW, Sheldon T. Investigating environmental determinants of injury and trauma in the Canadian North. Int. J. Environ. Res. Public Health 2014; 11: 1536–1548. 10.3390/ijerph110201536 24477214 PMC3945552

[8] World Health Organization. Global Report on Drowning: Preventing a Leading Killer (eds. Meddings, D. et al.)(WHO Press, Geneva, 2014). Available from: https://www.who.int/violence_injury_prevention/global_report_drowning/en/

[9] Giesbrecht GG. Cold Stress, Near Drowning and Accidental Hypothermia: A Review. Aviat. Space Environ. Med. 2000; 71: 733–752. 10902937

[10] Barss P. et al Drownings and Other Water-Related Injuries in Canada, 1991–2000. Module 2: Ice & Cold Water. Canadian Red Cross, 2006.

[11] 11. Funari, E, Giustini M. Annegamenti in Italia: epidemiologia e strategie di prevenzione. Rapporti ISTISAN 11/13 Istituto Superiore di Sanità, Roma, 2011. http://www.salvamentonettunoanzio.it/images/Progetti/ISTISAN/rapporto-istisan-2011-annegamenti-in-Italia.pdf

[12] Peden AE, Mahony AJ, Barnsley PD, Scarr, J. Understanding the full burden of drowning: a retrospective, cross-sectional analysis of fatal and non-fatal drowning in Australia. BMJ Open 2018: 8, e024868 10.1136/bmjopen-2018-024868 30473541 PMC6254411

[13] Lee TM, Markowitz M, Howe PD, Ko CY, Leiserowitz AA. Predictors of public climate change awareness and risk perception around the world. Nat. Clim. Change 2015; 5: 1014–1020.

[14] Benson BJ, Magnuson JJ, Jensen OP, Card VM, Hodgkins G, Korhonen J, et al Extreme events, trends, and variability in Northern Hemisphere lake-ice phenology (1855–2005). Clim. Change 2012; 112: 299–323.

[15] Magnuson JJ, Robertson DM, Benson BJ, Wynne RH, Livingston DM, Arai T, et al Historical trends in lake and river ice cover in the Northern Hemisphere. Science 2000; 289: 1743–1746. 10.1126/science.289.5485.1743 10976066

[16] Markus T, Stroeve JC, Miller J. Recent changes in Arctic sea ice melt onset, freezeup, and melt season length. J. Geophys. Res. 2009; 114: C12024, 10.1029/2009JC005436

[17] Brown LC, Duguay CR. The response and role of ice cover in lake-climate interactions. Progress in Physical Geography 2010; 34: 671–704.

[18] Stroeve JC, Serreze MC, Holland MM, Kay JE, Malanik J, Barrett AP. The Arctic’s rapidly shrinking sea ice cover: a research synthesis. Clim. Change 2012; 110: 1005–1027.

[19] Stewart KM, Magnuson JJ. Ice. in *Encyclopedia of Inland Waters* 2 (eds. Likens G. E. et al.) 664–670 (Elsevier, Boston, 2009).

[20] Block BD, Denfeld BA, Stockwell JD, Flaim G, Grossart HPF, Knoll LB, et al The unique methodological challenges of winter limnology. Limnol. Oceanogr.: Methods 2019; 17: 42–57.

[21] Harris I, Jones PD, Osborn TJ, Lister DH. Updated high-resolution grids of monthly climatic observations—the CRU TS3.10 Dataset. Int. J. Climatol. 2014; 34: 623–642.

[22] Strayer HD, Lucas DL, Hull-Jilly DC, Lincoln JM. Drowning in Alaska: progress and persistent problems. Int. J. Circumpolar Health 2010; 69: 253–264. 10.3402/ijch.v69i3.17627 20519089

[23] Weyhenmeyer GA, Livingstone DM, Meili M, Jensen O, Benson B, Magnuson JJ. Large geographical differences in the sensitivity of ice‐covered lakes and rivers in the Northern Hemisphere to temperature changes. Global Change Biol. 2011; 17: 268–275.

[24] Lepparanta M. Modelling the formation and decay of lake ice. *In:* D.G. George (ed.), The impact of climate change on European lakes. *Aquatic Ecology Series* 4 (2010).

[25] Lepparanta M, Terzhevik A, Shirasawa K. Solar radiation and ice melting in Lake Vendyurskoe, Russian Karelia. Hydrology Research 2010; 41: 50–62.

[26] Jakkila J, Lepparanta M, Kawamura T, Shirasawa K, Salonen K. Radiation transfer and heat budget during the ice season in Lake Paajarvi, Finland. Aquat Ecol. 2009; 43: 681–692.

[27] Lunetta P, Smith GS, Penttilä A, Sajantila A. Unintentional drowning in Finland 1970–2000: a population-based study. Int. J. Epidemiol. 2004; 33: 1053–1063. 10.1093/ije/dyh194 15218012

[28] Gustafsson T, Eriksson A. Off-road vehicle fatalities: a comparison of all-terrain vehicle and snowmobile accidents in Sweden. IATSS Research 2013; 37: 12–15.

[29] Fleischer NL, Melstrom P, Yard E, Brubaker M, Thomas T. The epidemiology of falling-through-the-ice in Alaska, 1990–2010. J. Public Health 2013; 36: 235–242. 10.1093/pubmed/fdt081 23995713 PMC5671923

[30] Schyllander J, Janson S, Nyberg C, Eriksson UB, Ekman DS. Case analyses of all children’s drowning deaths occurring in Sweden 1998–2007. Scand. J. Public Health 2013; 41: 174–179. 10.1177/1403494812471156 23282938

[31] Hedberg K, Gunderson PD, Vargas C, Osterholm MT, MacDonald KL. Drownings in Minnesota, 1980–85: a population-based study. Am. J. Public Health 1990; 80: 1071–1074. 10.2105/ajph.80.9.1071 2382743 PMC1404842

[32] Ahlm K, Saveman BI, Björnstig U. Drowning deaths in Sweden with emphasis on the presence of alcohol and drugs—a retrospective study, 1992–2009. BMC Public Health 2013; 13: 216 10.1186/1471-2458-13-216 23497055 PMC3610133

[33] Clemens T, Tamim H, Rotondi M, Macpherson AK. A population based study of drowning in Canada. BMC Public Health 2016; 16: 559 10.1186/s12889-016-3221-8 27411984 PMC4942881

[34] *Wisconsin Department of Natural Resources Snowmobile Safety and Enforcement Report* (2012–2013). https://dnr.wi.gov/files/pdf/pubs/le/LE0203_2013.pdf

[35] Kieboom JK, Verkade HJ, Burgerhof JG, Bierens JJ, van Rheenen PF, Kneyber MC, et al Outcome after resuscitation beyond 30 minutes in drowned children with cardiac arrest and hypothermia: Dutch nationwide retrospective cohort study. Brit. Med. J. 2015; 350: 1–10. 10.1136/bmj.h418 25670715 PMC4353310

[36] Slomine BS, Nadkarni VM, Christensen JR, Silverstein FS, Telford R, Topjian A, et al Pediatric cardiac arrest due to drowning and other respiratory etiologies: Neurobehavioral outcomes in initially comatose children. Resuscitation 2017; 115: 178–184. 10.1016/j.resuscitation.2017.03.007 28274812 PMC5429171

[37] Giles AR, Brooks Cleater L, McGuire-Adams T, Darroch F. Drowning in the social determinants of health: Understanding policy’s role in high rates of drowning in aboriginal communities in Canada. Aboriginal Policy Studies 2014; 3: 198–213.

[38] 38. Russian Emergency Situation Ministry. Rules of conduct on the ice and departure for the crossing (EMERCOM of Russia, accessed 24 April 2019)(2019). https://www.mchs.gov.ru/dop/info/individual/CHS_prirodnogo_haraktera/item/7263210/

[39] Kinney PL, Schwartz J, Pascal M, Petkova E, Le Tertre A, Medina S, et al Winter season mortality: will climate warming bring benefits? Environ. Res. Lett. 2015; 10: 1–13. 10.1088/1748-9326/10/6/064016 26495037 PMC4610409

[40] Sharma S, Meyer MF, Culpepper J, Yang X, Hampton S, Berger SA, et al Integrating perspectives to understand lake ice dynamics in a changing world. J. Geophys. Res.–Biogeosciences. In press.

[41] Royer MJS, Herrmann TM, Sonnentag O, Fortier D, Delusca K, Cuciurean R. Linking Cree hunters’ and scientific observations of changing inland ice and meteorological conditions in the subarctic eastern James Bay region, Canada. Clim. Change 2013; 119: 719–732.

