# Supplementary material for: Increased winter drownings in ice-covered regions with warmer winters
Source: PLoS One. 2020 Nov 18;15(11):e0241222. doi: 10.1371/journal.pone.0241222 (PMC7673519; doi:10.1371/journal.pone.0241222)
Supplement: S1 Fig — Winter drownings are shown as a percentage of total annual drowning deaths with median quartile, range, and extremes for the time period during which we collected records for all countries. The number of years of data collected with monthly drowning data is included above the box plot for each county. (DOCX) [file pone.0241222.s001.docx]

**S1 Fig.** **Boxplots summarizing drownings.** Winter drownings are shown as a percentage of total annual drowning deaths with median quartile, range, and extremes for the time period during which we collected records for all countries. The number of years of data collected with monthly drowning data is included above the box plot for each county.
