# Supplementary material for: Increased winter drownings in ice-covered regions with warmer winters
Source: PLoS One. 2020 Nov 18;15(11):e0241222. doi: 10.1371/journal.pone.0241222 (PMC7673519; doi:10.1371/journal.pone.0241222)
Supplement: S1 Table — For each of the 11 countries, we provide details on the types, sources and time periods of data that we used in this study. ICD Diagnosis codes are as follows: W69 (accidental drowning and submersion while in natural water), W70 (drowning and submersion following fall into natural waters), and W71 (and falling through the ice). (DOCX) [file pone.0241222.s002.docx]

**S1 Table. Detailed information on drowning data sources.** For each of the 11 countries, we provide details on the types, sources and time periods of data that we used in this study. ICD Diagnosis codes are as follows: W69 (accidental drowning and submersion while in natural water), W70 (drowning and submersion following fall into natural waters), and W71 (and falling through the ice).

| **Country** | **Details** | **Source** | **Time period** |
| --- | --- | --- | --- |
| Canada | As part of an on-going water-related fatality surveillance project, trained local data collectors enter each of the provincial and territorial Coroner’s and Medical Examiner’s offices annually to conduct structured reviews of the files for all unintentional water-related deaths. A structured questionnaire is used to collect data on demographics; cause of death; activity type and purpose of activity; and personal, equipment and environmental risk factors. Common documents in the files, and used to extract water-related fatality data, included: Coroner’s or Medical Examiner’s investigation statements, death certificates, police reports, hospital records, post-mortem/autopsy reports, and toxicology reports. For the purposes of this study, the numbers of drowning deaths in open water by month in each province and territory were extracted. | Drowning Prevention Research Centre (DPRC) database collected from the provincial and territorial Coroner and Medical Examiner Systems | Monthly.  January 1, 1991, to December 31, 2014 |
| Estonia | Data from codes W69 and W70. | Estonian Rescue Board | Monthly.  2010-2017 |
| Finland | Data from codes W69, W70, and W71. The Finnish Swimming Teaching and Lifesaving Federation obtains official statistics from the government and supplements them with media reports to obtain more information on when exactly the drowning occurred and under what conditions. | Finnish Swimming Teaching and Lifesaving Federation | Monthly. 1998-2016 |
| Germany | Data from code W69. Official statistics do not differ between “break-through-ice” related drowning and other drowning incidents. Hence, we assume that winter drownings (December – March) are associated with breaking through ice. | German Federal Statistics Office | Monthly. 2008-2016. |
| Italy | Data from codes W69 and W70. Data analysis was limited to the lake rich (> 650 lakes) Trentino-South Tyrol region,^44^ where distribution along the altitudinal gradient ensured that 90% of lakes are ice-covered in winter. | Italian Bureau of Statistics | Monthly. 2003-2015. |
| Japan | Drowning statistics from the Nagano Prefecture. | Nagano Police station | Monthly. 1999 to 2017 |
| Latvia | Data from codes W69. | Centre of Disease Prevention and Control | Monthly. 1996-2017. |
| Russia | This webpage was translated with the help of a Russian Colleague (Svetlana Serikova). | Ministry of the Russian Federation for Civil Defense, Emergencies and Elimination of Consequences of Natural Disasters | Winter.  2010-2014 |
| Sweden | The Swedish Rescue Society compiled drowning statistics from media reports, local and regional lifesaving societies, and police records. The compiled data are verified by official national records, including the National Board of Health’s Death Registry and the National Transportation Agency’s data on recreational watercraft accidents. | Swedish Rescue Society | Monthly. 1999-2016 |
| USA | Data from codes W69 and W70. USA data are summed for 14 northern states that experience winter ice cover on natural waters (Alaska, Connecticut, Iowa, Maine, Massachusetts, Michigan, Minnesota, Montana, New Hampshire, New York, North Dakota, South Dakota, Vermont, and Wisconsin). We extracted data for the winter months December, January, February, March, and April. | Centre for Disease Control | Monthly. 1999 to 2015 |
| USA - Minnesota | Highly detailed information was available, including where individuals drowned, how they drowned, and their ages. These detailed water accident reports and on ice-related fatalities are reported by county sheriff’s offices to the Minnesota Department of Natural Resources.. | Minnesota Department of Natural Resources | Daily.  1976-2018. |
